# Development and validation of a deep learning model using MR imaging for predicting brain metastases: an accuracy-focused study

**DOI:** 10.3389/fonc.2025.1657604

**Published:** 2025-09-23

**Authors:** Dan Shi, Meng Yang, Min Dong, Ning Xuan, Yinsu Zhu, Xiaoqiong Lv, Chao Xie, Fei Xia, Lingchun Xu, Qinglei Zhang, Na Yin

**Affiliations:** ^1^ Department of Radiology, Jiangsu Cancer Hospital, The Affiliated Cancer Hospital of Nanjing Medical University, Nanjing, China; ^2^ Department of Radiology, Nanjing Drum Tower Hospital, Affiliated Hospital of Medical School, Nanjing University, Nanjing, China; ^3^ The People’s Hospital of DanYang Oncology, Zhenjiang, China; ^4^ Independent Researcher, Los Angeles, CA, United States

**Keywords:** brain metastases, deep learning, artificial intelligence, diagnostic accuracy, magnetic resonance imaging

## Abstract

**Background:**

Brain metastases (BM), originating from extracranial malignancies, significantly threaten patient health. Accurate BM identification is crucial but labor-intensive manually. This study developed and validated a system for BM diagnosis, assessing its performance and stability.

**Methods:**

470 patients diagnosed with BM were divided into an 80% training set (n=379) and a 20% internal test set (n=91) using systematic sampling. An additional 172 patients were retrospectively enrolled for external validation. A comprehensive preprocessing pipeline was implemented. We developed a 3D U-Net model with a ResNet-34 backbone for BM prediction. MRI scans were resampled to 0.833 mm³ isotropic voxels, underwent skull stripping using SynthStrip, and were intensity-normalized via Z-score normalization. The model was trained on MRI scans paired with segmentation masks, utilizing ImageNet-pretrained encoder weights and a patch-based strategy (128×128×128 voxels).

**Results:**

The model maintained perfect specificity and AUCs across gender and age groups, with no significant differences in other metrics, confirming false positive exclusion unaffected by demographics. By cancer type: Internal testing showed significant difference of AUC (*p*<0.001) between lung cancer (n=74) and other cancers (n=17). The differences of other performance metrics were not statistically significant (*p*>0.13), though other cancers showed higher median F1/IoU/MCC. External validation showed other cancers (n=79) had significantly higher precision than lung cancer (n=93) (p<0.05). Lung cancer AUC (0.82) was significantly lower than other cancers (0.89) (p<0.001), suggesting need for sensitivity optimization; both maintained specificity=1.0000. Model time was significantly shorter than manual annotation (internal: 69s vs 113s; external: 66s vs 96s; both *p*<0.001), with high agreement.

**Conclusion:**

The model demonstrated strong robustness and perfect specificity across demographics. While showing cancer type dependency (requiring improved lung cancer sensitivity), its high efficiency (40%-50% time reduction) and generalization provide a solid foundation for clinical translation.

## 1 Introduction

Brain metastases (BM) are malignant tumors originating from extracranial primary tumors that metastasize to the brain parenchyma. Representing the most common type of intracranial tumor in adults, BM occur in approximately 10% to 40% of patients with solid tumors (1, 2). These lesions are predominantly located at the corticomedullary junction, characterized by insidious onset, rapid progression, and later manifestations including intracranial hypertension, neurological dysfunction, and epilepsy (3, 4). BM typically indicate advanced disease stage, and their incidence rises with prolonged patient survival. Consequently, early and precise detection is crucial for improving prognosis.

Current BM diagnosis relies heavily on neuroimaging, with magnetic resonance imaging (MRI) serving as the preferred modality due to its lack of ionizing radiation, superior soft-tissue resolution, and multi-sequence capabilities. Compared to computed tomography (CT), MRI demonstrates greater sensitivity for detecting posterior fossa lesions, multiple punctate metastases, and leptomeningeal disease. The typical MRI presentation is a ring-enhancing lesion on contrast-enhanced T1-weighted imaging (CE-T1WI) accompanied by significant peritumoral edema. However, traditional manual identification of multiple (especially small) metastatic foci is time-consuming and carries a high risk of missed diagnosis. Achieving efficient and accurate BM identification therefore remains a significant clinical challenge.

The advancement of artificial intelligence (AI) and radiomics in brain imaging critically depends on voxel-level image segmentation technology (5, 6). This technique partitions image regions based on features like intensity, shape, and texture to integrate targets, forming a fundamental prerequisite for computer-aided image analysis. The U-Net model, introduced by Ronneberger et al. (7), represents a major advancement. It efficiently utilizes limited annotated data, balances localization accuracy with contextual information, and offers advantages such as rapid segmentation, capacity for large image processing, and strong generalization. However, U-Net exhibits limitations, including an output size smaller than the input, dependence on specific tile sizes, constrained applicability of data augmentation techniques, and the requirement for manual loss function parameter tuning. To overcome the constraints of 2D processing, Cicek et al. (8) developed 3D U-Net. This architecture directly learns from sparsely annotated volumetric data to achieve dense 3D segmentation, supporting both semi-automatic and fully automatic workflows. By incorporating batch normalization and weighted loss functions, 3D U-Net significantly enhances performance while retaining the advantages of handling large datasets and robust generalization.

Previous research has developed various computer-aided diagnosis (CAD) systems for BM detection on MRI using diverse algorithms and sequences (9–13). Cho SJ et al. (10) conducted a comparative analysis of 12 recent studies, concluding that deep learning (DL) achieves BM detection rates comparable to classical machine learning approaches, with a lower per-case false positive rate. Despite ongoing CAD development, widespread clinical adoption faces hurdles. Most prior studies on BM detection rates are single-center retrospective analyses (9, 14–18), with the exception of a multicenter retrospective study by Xu J et al. (13). This reliance limits comprehensive evaluation of algorithmic stability and introduces potential selection bias. Furthermore, while previous models predict BM using MRI data (10, 16, 19), their robustness requires more thorough assessment.

This study aims to develop a 3D U-Net deep learning model based on the ResNet-34 backbone network. Through a systematic preprocessing pipeline and a multi-dimensional validation strategy, the model will achieve robust automatic segmentation of BM. Utilizing both internal and external datasets, stratified validation (by gender/age/cancer subtype subgroups) and model robustness testing will be conducted. Concurrently, the lesion detection performance between radiologists and the novel deep learning model for BM will be evaluated. The ultimate goal is to build an AI clinical decision-support tool to enhance both the precision and efficiency of brain tumor imaging diagnosis.

## 3 Manuscript formatting

## 4 Materials and methods

### 4.1 Study design and participants

This retrospective study was approved by the medical ethics committee and the patients’ informed consent was waived. A total of 470 patients diagnosed with BM in Jiangsu Cancer Hospital (Nanjing, China) from April 2022 to December 2024 included in our study were divided into 80% training set (379 cases) and 20% internal validation set (91 cases) using random sampling. In addition,172 patients diagnosed with BM at the Affiliated Drum Tower Hospital of Nanjing University Medical School (Nanjing, China) from February 2022 to September 2022 were used for external validation.

**Figure 1:**
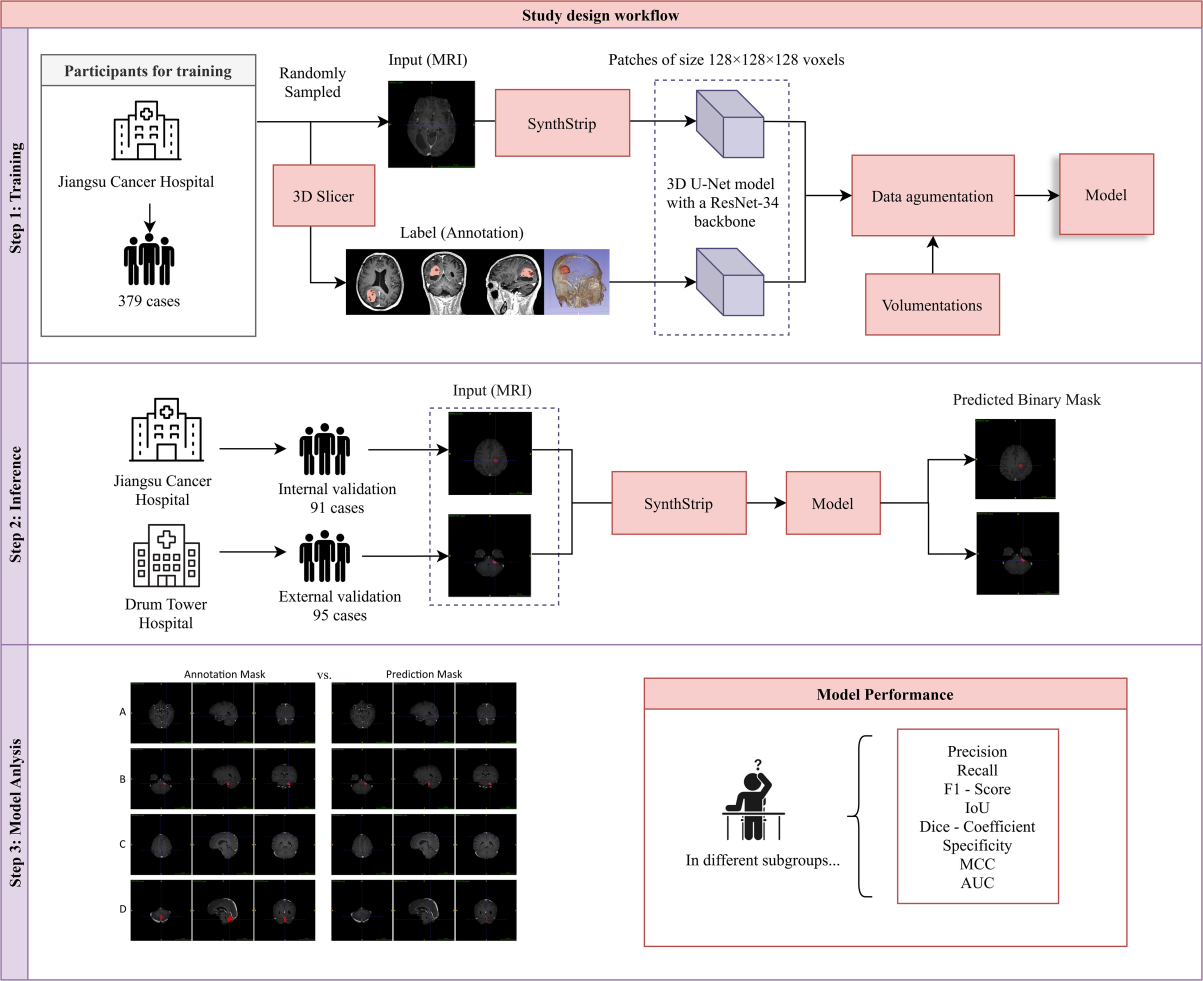
Study design workflow. Our study comprised three sequential phases: (1) Model Training: A 3D U-Net model with a ResNet-34 backbone was trained on the training dataset. (2) Inference: The model’s performance was evaluated using an internally curated testing set from the same hospital and an external validation set from another hospital. (3) Model Evaluation: Eight metrics were calculated to assess model performance, and a comparative analysis between model-based and manual delineations was conducted.

Participants who met following criteria were included in this study: (1) Patients were confirmed by clinical examinations to have brain metastases and had completed enhanced MRI scans of the brain. (2) Patients were aged 18 years or older and had complete clinical data. (3) The obtained MR images of patients were free of artifacts and distortion and had relatively high resolution. Meanwhile, to ensure the quality of the study, patients who meet any of following criteria would be excluded: (1) Patients with critical conditions and unstable vital signs. (2) Patients who were unable to tolerate MRI examinations and had only completed plain MRI scans without being able to undergo enhanced scans. (3) Patients with other serious cardiovascular and cerebrovascular diseases. (4) Patients with contraindications for MRI examinations, such as those with implanted cardiac pacemakers. (5) Patients whose imaging data have problems such as severe artifacts, noise, or motion blur (**Figure 1**).

### 4.2 MRI contrast-enhanced scan

The 3.0T superconducting MRI machine produced by Philips is selected. Instruct the patient to take the supine position and keep the head stable. A 32-channel head quadrature coil is used to collect data. The contrast injection method is to inject gadopentetate dimeglumine (Gd - DTPA) through the cubital vein at an injection flow rate of 1 mL/s and a dose of 0.25 mL/kg. Scanning and collecting data were performed after a delay of 3 minutes. The sequence settings for the contrast-enhanced 3D - T1WI scan were as follows: the sequence was 3D - FFE, the matrix was 256×256, the slice spacing/slice thickness was 1 mm/0 mm, the TR/TE (ms) was 6.6/3.0, the FOV was 240×240, and the voxel size was 1 mm * 1 mm * 1 mm.

### 4.3 Image selection and delineation

Three magnetic resonance physicians with more than 8 years of work experience reviewed the films independently, evaluated all the metrics of participants, and selected the magnetic resonance images that met the MRI imaging characteristics of brain metastases. They then manually outlined the enhanced metastatic lesions using software. The enhancement patterns could be divided into standardized uniform nodular enhancement, ring-shaped enhancement, and irregular enhancement. Standardized uniform nodular enhancement described that during the contrast-enhanced scan, both the interior and the edge of the nodular lesion shown uniform and significant enhancement. Ring-shaped enhancement meant that the lesion appeared nodular. During the contrast-enhanced scan, the central part of the lesion was not enhanced, and the enhancement of the edge was incomplete, forming a ring shape. Irregular enhancement was manifested as follows: although the lesion had nodular features, the enhancement effect was not uniform, or the shape of the lesion itself was irregular, and the enhancement patterns were diverse, which might be partially uniform and partially non-uniform.

## 5 Method

### 5.1 Implementation

**Figure 2:**
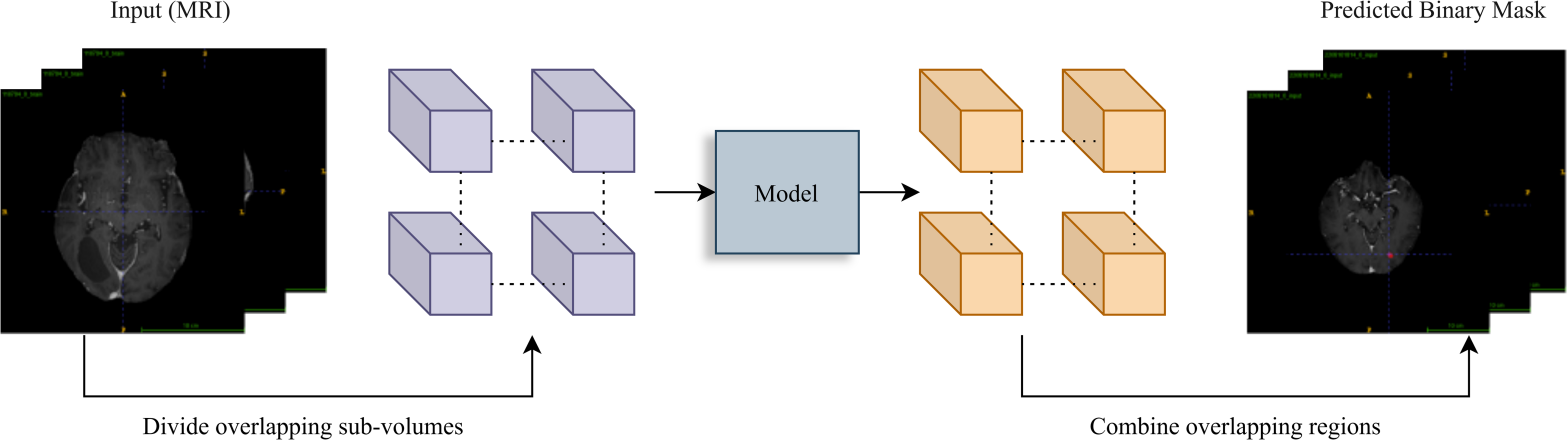
Inference workflow. A sliding window (stride=64) divides the input MRI volume into overlapping sub-volumes. Each is processed by the trained model for prediction. Overlapping region outputs are averaged to reconstruct the final binary tumor segmentation mask, ensuring smooth transitions.

To ensure robust and high-quality processing for model training and evaluation, we implemented a comprehensive preprocessing pipeline and developed a 3D U-Net model with a ResNet-34 backbone tailored for volumetric medical image segmentation. The preprocessing steps focused on standardizing the spatial resolution of MRI scans, normalizing intensity distributions, and implementing quality control measures. The 3D U-Net model utilized a pretrained encoder for efficient feature extraction, a patch-based training strategy to handle large MRI volumes, and data augmentation techniques to enhance model robustness and generalizability (20) (**Figure 2**).

### 5.2 Training data

The training data consisted of MRI scans paired with segmentation masks to enable supervised learning for the segmentation task. Preprocessing began with resampling the MRI scans to a uniform voxel size of 0.833 mm³. This standardization minimized variability caused by differences in scanner resolution and acquisition parameters. An automated verification step was employed to ensure alignment between the MRI scans and their corresponding segmentation masks, flagging mismatched pairs for manual review. Brain extraction was performed using SynthStrip (17), which removed non-brain tissues and reduced computational overhead by focusing on relevant brain regions. Intensity normalization followed, employing Z-score normalization to standardize voxel intensities, mitigating inter-subject variability due to scanner settings or patient-specific factors. Quality control was conducted by visualizing intensity histograms using matplotlib, ensuring consistency and reliability in the preprocessing pipeline.

### 5.3 Inference data for generating segmentation masks

The inference data comprised both internal and external datasets. The internal test set included 91 cases drawn from the same distribution as the training data, providing a baseline for performance assessment under known conditions. The external validation set consisted of 95 cases from an independent dataset, offering a robust evaluation of the model’s generalizability. Ground-truth segmentation masks were provided for both datasets, enabling a direct comparison of predicted and actual segmentations. This multi-faceted evaluation ensured that the model’s performance was rigorously assessed across diverse scenarios.

## 6 Experiment

### 6.1 Training

The 3D U-Net model with a ResNet-34 backbone was optimized for volumetric segmentation. The encoder, initialized with pretrained ImageNet weights, accelerated convergence and enhanced feature extraction efficiency. A patch-based training strategy was employed, dividing MRI scans into patches of size 128×128×128 voxels to accommodate the memory constraints posed by the large data volumes. Data augmentation was applied using volumentations, incorporating techniques such as left-right flipping, elastic deformations, and gamma adjustments. These augmentations simulated anatomical and imaging variability, boosting the model’s robustness to unseen data. The hybrid loss function, which combined Dice loss and binary cross-entropy with equal weighting, was chosen to balance pixel-wise accuracy with overlap-based evaluation metrics. Optimization was carried out using the Adam optimizer with a learning rate of 0.0001 and parameters β1 = 0.9, β2 = 0.999, ∈=1×10−7β1​=0.9, β2​=0.999, ∈=1×10−7. To prevent overfitting, a dropout rate of 0.5 was applied in the decoder layers. A batch size of 4 was used, constrained by the memory limitations of the NVIDIA RTX 4090 GPU with 64 GB of system RAM. The training process was implemented in TensorFlow and Keras.

### 6.2 Inference: generating segmentation masks

The trained 3D U-Net model was applied to both internal and external datasets to generate segmentation masks. MRI scans were preprocessed to align with the model’s input requirements, including brain extraction and normalization. A patch-based inference method was adopted, employing overlapping patches with a 50% overlap (stride = 64 voxels). Predictions from overlapping patches were averaged to minimize boundary artifacts and produce smoother segmentation results. Binary segmentation masks were generated by thresholding the model’s outputs at 0.5. Performance metrics, including Area Under the Curve (AUC), Dice coefficient, F1-score, Intersection over Union (IoU), Matthew’s correlation coefficient (MCC), precision, recall, and specificity were calculated using scikit-learn. These metrics were aggregated and analyzed using pandas for statistical analysis.

### 6.3 Statistical analysis

For the descriptive analysis, continuous variables with normal distribution were presented as mean and standard deviation (SD), and those with a non-normal distribution were presented as median and interquartile range (IQR). Categorical variables were summarized as counts (n) with corresponding percentages (%). Continuous variables were assessed for statistical differences using two-sample t test or Mann–Whitney U tests. Categorical variables were evaluated for differences using the χ2 test. Subgroup analyses were conducted to identify the population in which this model is most suitable for delineating lesions between each subgroup. The internal testing set and the external validation set were both stratified by age (< 60 years and >= 60 years), gender, and primary cancer type (lung cancer and other cancer). For metrics (precision, recall, F1 score, IoU, dice coefficient, specificity, and MCC), differences between each subgroup were assessed by employing Mann–Whitney U tests, with corresponding 95% confidence intervals (CIs) estimated. The comparison of AUC between subgroups was performed by using DeLong test. Additionally, the differences of time cost between model-based delineation and manual annotation were also compared by using Mann–Whitney U tests in both internal testing set and external validation set. All statistical analyses were performed using R version 4.4.1. A P-value of less than 0.05 was considered statistically significant.

## 7 Result

### 7.1 Patient characteristics

This retrospective study included 91 patients in the test set and 172 patients in the validation set. The median baseline ages in the test and validation sets were 59.4 ± 10.2 and 57.4 ± 12.6 years, respectively. Demographic and clinical characteristics, including age and gender, were comparable between the two cohorts, with no statistically significant differences observed. Selection bias was present regarding cancer type, showing a statistically significant difference. The clinical characteristics of the patients are summarized in **Table 1**.

**Table 1:** Characteristics of patients in testing set and validation set.

Characteristics	Testin set(*N* = 91)	Validation set(*N* = 95)	*p*-value
Age	59.4 ± 10.2	57.4 ± 12.6	0.173
<60	49	82	0.411
≥60	42	90	
Gender			0.136
Male	5	84	
Female	37	88	
Cancer type			<0.001
Lung cancer	74	93	
Other type	17	79	

### 7.2 Model performance evaluation

**Table 2** describes the case characteristics in the internal test set and external validation set. In our study, model performance on both the internal test set and external validation set was evaluated by calculating Precision, Recall, F1 Score, Intersection over Union (IoU), Dice Coefficient, Specificity, Matthews Correlation Coefficient (MCC), and the Area Under the Receiver Operating Characteristic Curve (AUC). Results were summarized using median and interquartile range (IQR) (**Table 2**). The results showed that the model achieved an AUC of 0.89 (IQR 0.79–0.93) on the internal test set and 0.82 (IQR 0.67–0.90) on the validation set, indicating good overall diagnostic capability. Furthermore, Precision was >0.93 in both the internal test set and external validation set, suggesting strong discriminative ability, with the external validation set showing higher precision than the test set. Recall decreased from 0.78 (IQR 0.57–0.86) in the test set to 0.64 (IQR 0.34–0.81) in the validation set, accompanied by a synchronous decline in F1 Score (0.82 vs. 0.75), indicating moderate model robustness. Specificity reached 1.000 (IQR 1.000–1.000) in both the test and validation sets, signifying theoretically optimal ability to exclude negative samples. MCC decreased to 0.77 (IQR 0.55–0.86) in the validation set, indicating good classification reliability; the Dice Coefficient also decreased synchronously to 0.75 (IQR 0.49–0.85).

**Table 2:** Summary descriptive table by groups of datasets.

Metrics	Testing set (*N* = 91)	Validation set (*N* = 172)
Precision	0.926(0.831,0.966)	0.935(0.858,0.967)
Recall	0.782(0.567,0.861)	0.638(0.342,0.806)
F1 Score	0.821(0.674,0.882)	0.750(0.495,0.852)
IoU	0.697(0.508,0.789)	0.600(0.329,0.743)
Dice Cofficient	0.821(0.674,0.882)	0.750(0.495,0.852)
Specificity	1.000(1.000,1.000)	1.000(1.000,1.000)
MCC	0.832(0.698,0.883)	0.768(0.549,0.857)
AUC	0.891(0.793,0.930)	0.819(0.671,0.903)

**Figure 3:**
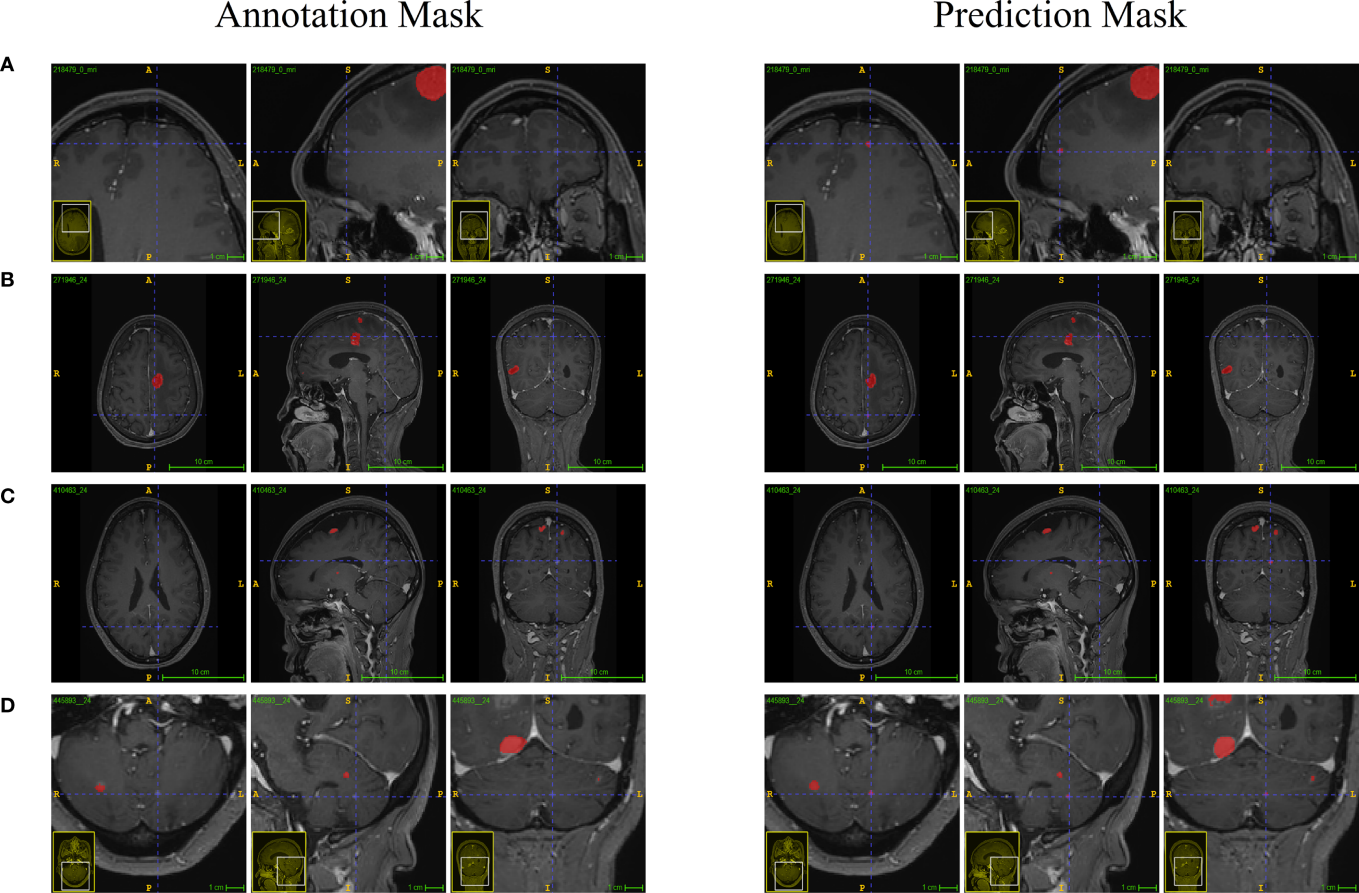
Manual identification and model identification of the lesion legend.

As shown in **Figure 3a** comparison between clinicians and the model in identifying brain metastatic lesions was conducted, both for the test set and the validation set. The clinical validation data indicated that compared with the manual interpretation by clinicians, the number of lesions detected by this model was significantly higher, effectively reducing the rate of missed detections, highlighting its potential value in improving the accuracy of clinical diagnosis.

### 7.3 Model performance comparative analysis

To comprehensively evaluate model robustness, this study conducted stratified validation across three dimensions (gender, age, and primary cancer type). Additionally, stability assessments were performed on both datasets (internal test set and external independent validation set).

**Figure 4:**
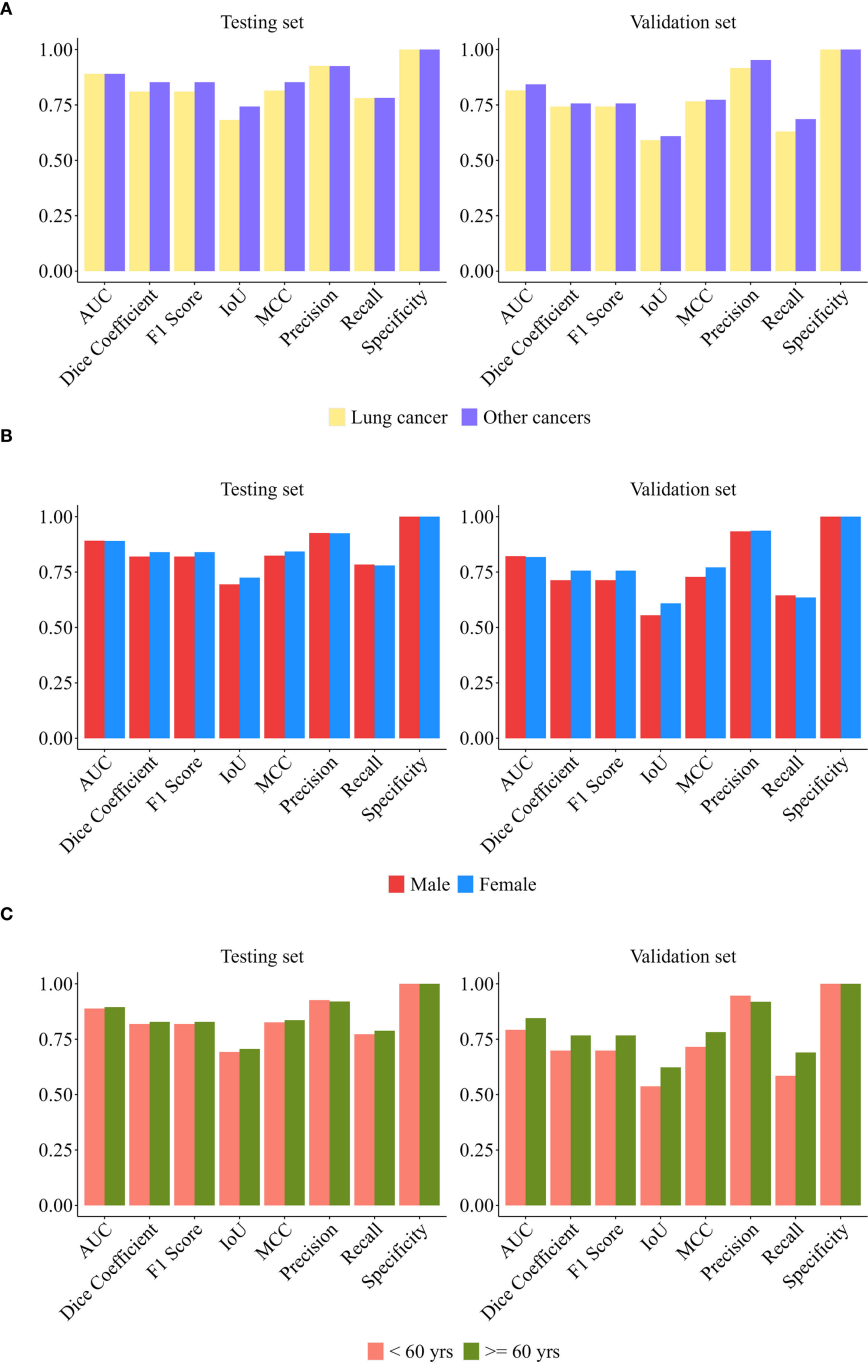
Training vs. validation set comparisons by subgroup. **(A)** Cancer type **(B)** Gender **(C)** Age.

**Figure 4** systematically presents the multi-dimensional evaluation results of the model in the internal test set and the external validation cohort.

In the internal test set (n = 91): Our model demonstrated superior predictive performance in males, patients < 60 years old, and patients with other cancer compared with the other groups. The AUC was 0.89 for males, 0.91 for patients younger than 60 years old, and 0.91 for patients with other cancer type. DeLong tests indicated that these between-group differences were statistically significant (all p < 0.001). Subgroup analysis by cancer type (lung cancer n = 74, other cancers n = 17) revealed: There was no significant difference of other metrics between groups (p > 0.05), and its high specificity (1.0000) and stable AUC (> 0.88) supported generalization ability. The median of other cancer group comprehensive indicators (F1/IoU/MCC) was higher, suggesting that the model may have better discrimination ability for non-lung cancer malignancies. Subgroup analysis by gender (male n = 54, female n = 37) showed that apart from the AUC, the differences of other performance metrics between subgroups were not statistically significant (p > 0.10), and the model performance was highly consistent; Specificity reached 1.0000, indicating that the model’s ability to exclude false positives was extremely strong. Age subgroup analysis (< 60 years old n = 49, ≥ 60 years old n = 42) indicated: The age factor did not significantly change the model performance metrics (p > 0.20); The lower limit of the recall rate distribution in the ≥ 60 years old group (Q1 = 0.5540) suggested that the sensitivity needed to be optimized to reduce the risk of missed diagnosis in the elderly.

In the external validation set (n = 172), the prediction model achieved better comprehensive performance in the male subgroup (AUC = 0.86), the subgroup aged ≥60 years (AUC = 0.90), and the subgroup with other cancers (AUC = 0.89). DeLong tests indicated that the differences of AUC between subgroups were statistically significant (p < 0.001). The subgroup analysis by cancer type (lung cancer n = 93, other cancers n = 79) showed that the precision of the other cancer group was significantly higher (p < 0.001), indicating that the model has a discriminative advantage in identifying non-lung cancer malignancies. Both subgroups maintained a perfect specificity (1.0000), verifying the model’s generalization ability in controlling false positives; the differences in the remaining indicators between the lung cancer group and the non-lung cancer group were not statistically significant (all p > 0.26), suggesting that the model performance is cancer type-dependent. The comprehensive performance of the lung cancer group (AUC = 0.82) was significantly lower than that of the other cancer group, indicating that the model needs to be further optimized for its sensitivity to lung cancer. The statistical analysis grouped by gender (male n = 84; female n = 88) showed that except for AUC, the differences in evaluation indicators between the male group and the female group were not statistically significant (all p > 0.52), indicating that the model performance is stable across the gender dimension. The high specificity (1.0000) and stable AUC value (> 0.84) remained consistent in the cross-center validation, reflecting the model’s good robustness. In the age subgroup analysis (< 60 years old n = 82, ≥ 60 years old n = 90), apart from AUC, the differences in other indicators between the two age groups were not statistically significant (p > 0.05), indicating that the model performance was highly consistent across different age groups; the perfect specificity (1.0000) and stable AUC value (> 0.82) further confirmed the reliability of the model in excluding false positives.

To further demonstrate the clinical practicability of this model, we conducted a comparative analysis of the time spent on model-based depiction and manual lesion annotation. The results showed that, in both datasets, the time required for model-based depiction was significantly less than that of manual annotation (both P < 0.001) (**Table 3**), and it maintained a comparable consistency with manual depiction (**Figure 3**). In the internal test set, the median time of the model was 69s (IQR 68 - 69), while the median time of manual annotation was 113 seconds (IQR 90 - 151). In the external validation set, the median time of the model was 66s (IQR 65 - 68), while the median time of manual annotation was 96s (IQR 78 - 113).

**Table 3:** Comparison of time consuming in model-based and manual annotation in internal testing set and external validation set.

Dataset	Time, s		Z	P
	Model	Annotation		
Testing	69 (68,69)	113 (90,151)	-11.672	<0.001
Validation	66 (65, 68)	96 (78,113)	-14.143	<0.001

## 8 Discussion

In this multicenter study, we developed a 3D U-Net deep learning model based on the ResNet-34 backbone network for the detection of brain metastases (BM) in 3D CET1WI MRI images of a large number of patients. Additionally, we conducted a multi-reader evaluation aimed at quantifying the impact of the BM-assisted system on reading time and lesion detection efficacy. Through multi-dimensional stratified validation, this study systematically evaluated the robustness of the model on the internal test set and the external independent validation set. The results showed that the model demonstrated perfect specificity (Specificity = 1.0000) for both male and female patients, strongly confirming its ability to exclude false positive results regardless of gender factors. Similarly, in the age stratification analysis (<60 years vs. ≥60 years), the core efficacy indicators (such as AUC, specificity) were highly consistent between the two age groups, supporting its universality in patients of different ages. In the cancer type dimension, the model performed comparably in the internal test set for lung cancer and other malignant tumors overall; while in the external validation set, it showed a significantly higher precision rate for non-lung cancer malignant tumors (the “other cancer” group). Although both groups maintained perfect specificity, the comprehensive performance indicators of the lung cancer group in the external validation were relatively lower, suggesting that the model performance may have some cancer type dependence. The model yielded better predictive performance when applied to the subgroups of males and non-lung cancer patients. Future optimization should focus on improving the model’s sensitivity for detecting lung cancer lesions. Overall, this model significantly improved the efficiency in detecting BM (with much less time than manual annotation), and its excellent robustness demonstrated a good potential for clinical translation applications.

BMs are the most common intracranial malignant tumors, with an incidence of approximately 20%-40% (1, 4, 21). BM often leads to severe neurological lesions and shortens survival time, and its early diagnosis is closely related to clinical decisions and patient prognosis (22). MRI, with its high soft tissue resolution and rich scanning sequences, has become the main examination method for BM. Although MRI can provide various imaging features, manual visual diagnosis often fails to cover all effective features and ignores the complexity of tissue cells, resulting in difficult improvement of detection accuracy to the application level. Therefore, developing an automated brain tumor segmentation technology to achieve high-precision and repeatable measurement of tumor substructures, replacing the current manual basic assessment, has become an urgent need to supplement BM diagnosis. Fajam et al. (9) in a prospective single-center study included 29 patients and developed a set of uneven 3D spherical template to detect brain metastases in Gd-enhanced T1WI imaging, achieving a sensitivity of 93.5% and an intracranial false positive rate of 0.024. However, the system in their study was not evaluated clinically. Lu SL et al. (12) demonstrated through a random multi-reader multi-case study of 10 patients (a total of 23 tumors) that the automatic detection and segmentation technology based on deep neural networks (ABS system) can improve the accuracy and efficiency of tumor contour delineation and reduce the differences among doctors. However, this study focused more on tumor segmentation rather than detection, and did not analyze reading time. Xue J et al. (13) retrospectively included 1625 patients from three centers and constructed a model for detecting and segmenting brain metastases, although the sensitivity, specificity, and Dice coefficient of the model were evaluated, the reading time of the model compared with manual recognition was not assessed. Unlike previous studies, this research employs a 3D U-Net deep learning model based on the ResNet-34 backbone to detect brain metastases on 3D enhanced T1-weighted MRI images of a large number of patients. Through multi-dimensional hierarchical validation on the internal test set and external validation set, the BM segmentation precision of this 3D U-Net model reaches 92.6% and 93.5% respectively. Meanwhile, the specificity of our model in the internal test set and external validation set is also very outstanding. Compared with previous studies, the model in this research has higher precision and specificity (1, 23). Amemiya S et al. developed a combined algorithm based on feature fusion and single detector (24), which has a high overall sensitivity and specificity for brain metastases without reducing the positive predictive value, thereby improving the detection rate of small lesions. Huang et al. (25) proposed a deep learning model based on volume-level sensitivity and specificity, which also shows high sensitivity and accuracy for BM detection.

One of the most commonly used network architectures is the so-called U-Net (26, 27). Recently, Pfluger I et al. developed a system based on artificial neural networks (28) using the nnU-Net method to segment meningiomas from 308 patients (29). Bousabarah K et al. implemented traditional U-Net and an improved U-Net with multiple outputs to achieve automatic separation of meningiomas (30). Additionally, Gong J et al. proposed an integrated learning model based on deep learning and image-based radiomics to improve the prediction ability of the risk of brain metastasis in patients with advanced non-small cell lung cancer within three years (31). By applying the deep residual U-Net model, each pulmonary tumor can be automatically and accurately segmented, combined with CT image-based radiomics and clinical features. This improves the performance of predicting the risk of meningiomas. We also found that the quality of automatic segmentation is very high (0.8≥DSC≥0.6) in both the internal test set and external validation set, indicating that the model can provide more reliable image segmentation for brain metastases. Zhu Genste et al. trained a deep learning model for detecting and three-dimensional segmenting brain metastases in non-small cell lung cancer (32). Compared with manual segmentation, the DSC consistency coefficient reached 0.72.

Most previous studies used classical machine learning or deep learning and multiple sequences to detect or segment brain metastases based on the number and size of the lesions (33, 34). Although these studies mostly evaluated overall sensitivity, accuracy, and per capita false positive rate, they covered various different primary tumor subtypes. Although multi-modal modalities have significant advantages, the additional scanning time and sequence availability costs may hinder their widespread clinical application. Our method uses only the CE TIWI sequence and can detect brain metastases with a precision of over 80%. Given the trade-off between precision and specificity, our model may be more suitable for wide clinical application. Unlike previous studies, this research validates the model through multi-dimensional hierarchical validation, firmly confirming that the model is not affected by factors such as gender and age, supporting its universality in patients of different ages and genders. Although this model shows certain type-dependent performance in the cancer type dimension for the identification of lung cancer and other cancers, its efficiency has significantly improved and the overall performance is quite good, demonstrating good potential for clinical translation applications.

Our research indicates that the time required for lesion identification based on the model is significantly shorter than that of manual identification, effectively improving the efficiency of clinical diagnosis. Compared with other technologies (33, 34), this study has several unique features. Firstly, our research includes training sets and validation sets to verify our system, which is more persuasive than previous studies. Secondly, to evaluate the stability of the model, this study conducts stratified validation from multiple dimensions (gender, age, and cancer type), and the results show that the internal and external validation sets of the model exhibit excellent robustness, showing good universality for patients of different genders and different age groups. The model has a certain type-dependent performance for cancer. Moreover, the efficiency of this model in identifying BM is significantly higher than manual annotation. Combined with its excellent robustness, it has good potential for clinical translation applications.

This study also has potential limitations. Firstly, the limiting factor for the model to achieve higher maturity is the small number of samples of BM patients included in the training and validation sets. Secondly, our model has some false negative results in identifying BM; BM and blood vessels may appear as nodules or high-signal spots on CE T1WI, and the latter may be mistakenly regarded as BM. Finally, it is necessary to validate our model on a larger dataset and further verify its stability in multiple centers. Subsequently, a stratified analysis of lesion size will be conducted to prevent missed diagnoses of lesions.

In summary, our study shows that the 3D U-Net model demonstrates perfect specificity and robustness across transgender and age groups, with slight differences in cancer types (the sensitivity of lung cancer needs to be optimized). Overall, it is highly efficient and accurate, and has significant clinical translational value. Based on our multi-center evaluation, this system helps radiologists with different levels of experience achieve higher detection specificity and precision.

## Data Availability

The raw data supporting the conclusions of this article will be made available by the authors, without undue reservation.
