# Supplementary figures and images for: Development and validation of a deep learning model using MR imaging for predicting brain metastases: an accuracy-focused study
Source: Front Oncol. 2025 Sep 23;15:1657604. doi: 10.3389/fonc.2025.1657604 (PMC12500715; doi:10.3389/fonc.2025.1657604)

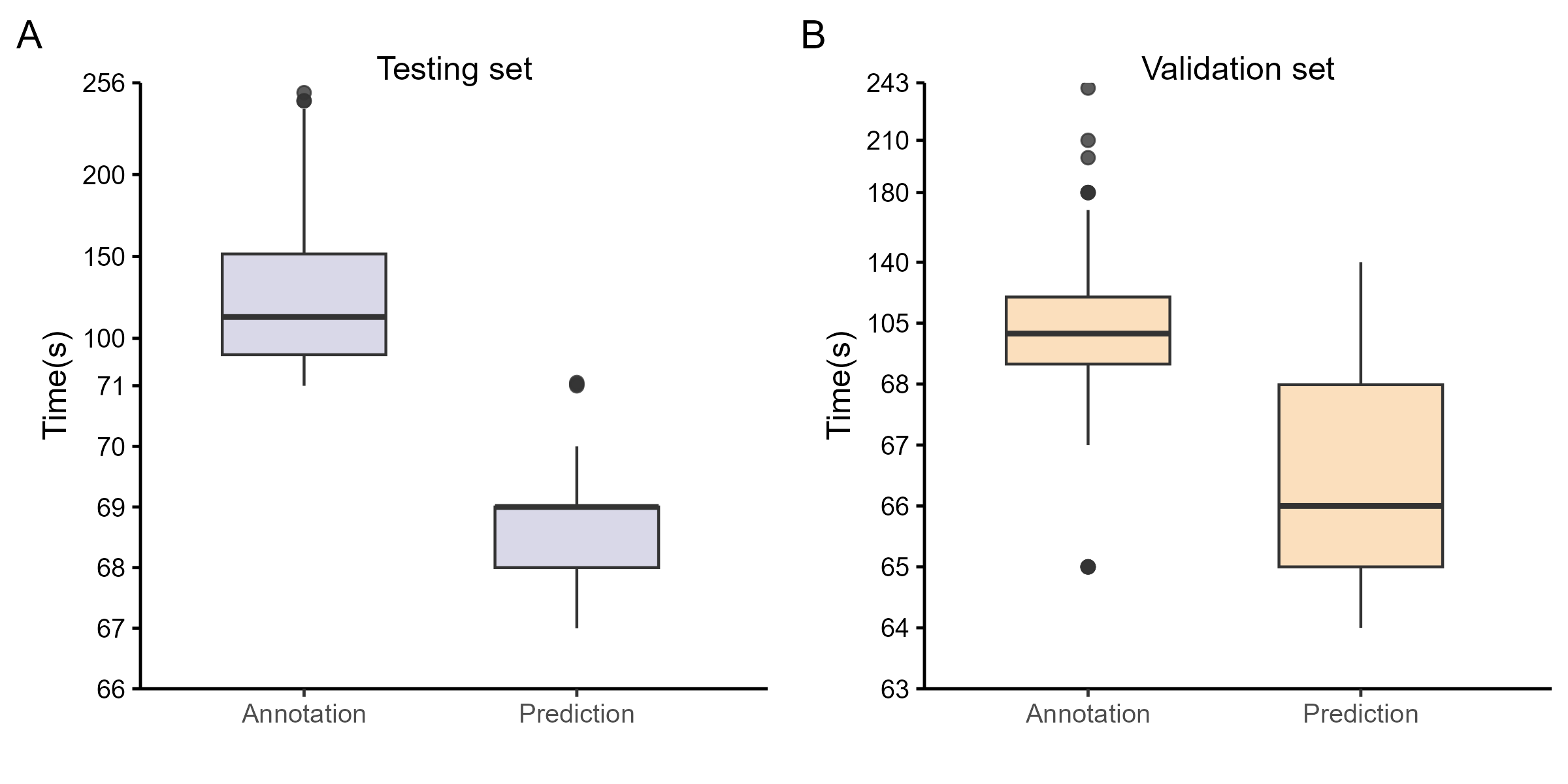

Supplement: Supplementary file 1 [file Image1.tiff]
