# Supplementary material for: Development and validation of a deep learning model using MR imaging for predicting brain metastases: an accuracy-focused study
Source: Front Oncol. 2025 Sep 23;15:1657604. doi: 10.3389/fonc.2025.1657604 (PMC12500715; doi:10.3389/fonc.2025.1657604)
Supplement: Supplementary file 2 [file Table1.docx]

Subgroup analyses

Comparison of area under curve between subgroups in testing set

| subgroup | AUC | p-value |
| --- | --- | --- |
| Gerden |  | <0.001 |
| Male | 0.8926 (0.8921, 0.8932) |  |
| Female | 0.8897 (0.889, 0.8904) |  |
| Age |  | <0.001 |
| < 60 yrs | 0.9134 (0.9129 ,0.9139) |  |
| >= 60 yrs | 0.8538 (0.853 ,0.8545) |  |
| Primary cancer type |  | <0.001 |
| Lung cancer | 0.8816 (0.8811, 0.8821) |  |
| Other cancer | 0.9124 (0.9117, 0.9131) |  |

Comparison of area under curve between subgroups in validation set

| subgroup | AUC | p-value |
| --- | --- | --- |
| Gerden |  | <0.001 |
| Male | 0.8602 (0.8598, 0.8606) |  |
| Female | 0.846 (0.8456, 0.8463) |  |
| Age |  | <0.001 |
| < 60 yrs | 0.8205 (0.8202, 0.8209) |  |
| >= 60 yrs | 0.9003 (0.9, 0.9007) |  |
| Primary cancer type |  | <0.001 |
| Lung cancer | 0.8156 (0.8152, 0.8159) |  |
| Other cancer | 0.8912 (0.8908, 0.8915) |  |

Summary descriptives table for Male and Female in testing set

| Metrics | **Male (n=54)** | **Female (n=37)** | **95% CI** | **p-value** |
| --- | --- | --- | --- | --- |
| Precision | 0.9263 (0.8275, 0.9744) | 0.9255 (0.8383, 0.9646) | (-0.0344, 0.0312) | 0.9903 |
| Recall | 0.7837 (0.5540, 0.8696) | 0.7796 (0.6114, 0.8425) | (-0.0406, 0.0937) | 0.4191 |
| F1 Score | 0.8195 (0.6720, 0.8824) | 0.8403 (0.6821, 0.8767) | (-0.0431, 0.0594) | 0.7343 |
| IoU | 0.6942 (0.5060, 0.7896) | 0.7245 (0.5176, 0.7805) | (-0.0651, 0.0881) | 0.7343 |
| Dice-Coefficient | 0.8195 (0.6720, 0.8824) | 0.8403 (0.6821, 0.8767) | (-0.0431, 0.0594) | 0.7343 |
| Specificity | 1.0000 (1.0000, 1.0000) | 1.0000 (1.0000, 1.0000) | (0, 0) | 0.1811 |
| MCC | 0.8239 (0.6986, 0.8845) | 0.8436 (0.6993, 0.8799) | (-0.0399, 0.0566) | 0.7101 |

Summary descriptives table for age < 60 yrs, and >= 60 yrs in testing set

| Metrics | **<60 Yrs (n=49)** | **>=60 Yrs (n=42)** | **95% CI** | **p-value** |
| --- | --- | --- | --- | --- |
| Precision | 0.9264 (0.8366, 0.9740) | 0.9205 (0.8318, 0.9594) | (-0.0184, 0.0469) | 0.3641 |
| Recall | 0.7724 (0.6114, 0.8762) | 0.7886 (0.5540, 0.8555) | (-0.0658, 0.0626) | 0.9176 |
| F1 Score | 0.8185 (0.7031, 0.8825) | 0.8280 (0.6105, 0.8767) | (-0.0445, 0.0572) | 0.8735 |
| IoU | 0.6928 (0.5422, 0.7897) | 0.7065 (0.4393, 0.7804) | (-0.0656, 0.0855) | 0.8735 |
| Dice-Coefficient | 0.8185 (0.7031, 0.8825) | 0.8280 (0.6105, 0.8767) | (-0.0445, 0.0572) | 0.8735 |
| Specificity | 1.0000 (1.0000, 1.0000) | 1.0000 (1.0000, 1.0000) | (0, 0) | 0.2262 |
| MCC | 0.8263 (0.7346, 0.8826) | 0.8361 (0.6301, 0.8796) | (-0.0416, 0.0534) | 0.836 |

Summary descriptives table for lung cancer, and other cancer in testing set

| Metrics | **Lung cancers (n=74)** | **Other cancers(n=17)** | **95% CI** | **p-value** |
| --- | --- | --- | --- | --- |
| Precision | 0.9263 (0.7580, 0.9658) | 0.9255 (0.8683, 0.9650) | (-0.0647, 0.0251) | 0.436 |
| Recall | 0.7813 (0.5410, 0.8429) | 0.7817 (0.7480, 0.8841) | (-0.1377, 0.0266) | 0.2013 |
| F1 Score | 0.8107 (0.6105, 0.8783) | 0.8525 (0.8106, 0.9059) | (-0.1305, 0.0036) | 0.0723 |
| IoU | 0.6816 (0.4393, 0.7830) | 0.7429 (0.6815, 0.8279) | (-0.1824, 0.0054) | 0.0723 |
| Dice-Coefficient | 0.8107 (0.6105, 0.8783) | 0.8525 (0.8106, 0.9059) | (-0.1305, 0.0036) | 0.0723 |
| Specificity | 1.0000 (1.0000, 1.0000) | 1.0000 (1.0000, 1.0000) | (0, 0) | 0.2942 |
| MCC | 0.8145 (0.6234, 0.8799) | 0.8529 (0.8118, 0.9058) | (-0.1212, 0.0029) | 0.079 |

Summary descriptives table for Male and Female in validation set

| Metrics | **Male (n=84)** | **Female (n=88)** | **95% CI** | **p-value** |
| --- | --- | --- | --- | --- |
| Precision | 0.9339 (0.8279, 0.9672) | 0.9364 (0.8677, 0.9674) | (-0.0253, 0.0117) | 0.5229 |
| Recall | 0.6447 (0.3417, 0.8267) | 0.6356 (0.3432, 0.7959) | (-0.0525, 0.0958) | 0.636 |
| F1 Score | 0.7138 (0.4975, 0.8556) | 0.7568 (0.4936, 0.8478) | (-0.0489, 0.0599) | 0.7792 |
| IoU | 0.5550 (0.3311, 0.7476) | 0.6087 (0.3277, 0.7358) | (-0.0628, 0.0815) | 0.7792 |
| Dice-Coefficient | 0.7138 (0.4975, 0.8556) | 0.7568 (0.4936, 0.8478) | (-0.0489, 0.0599) | 0.7792 |
| Specificity | 1.0000 (1.0000, 1.0000) | 1.0000 (1.0000, 1.0000) | (0, 0) | 0.8976 |
| MCC | 0.7283 (0.5481, 0.8596) | 0.7719 (0.5514, 0.8496) | (-0.0486, 0.0551) | 0.8052 |

Summary descriptives table for age < 60 yrs, and >= 60 yrs in validation set

| Metrics | **<60 Yrs (n=82)** | **>=60 Yrs (n=90)** | **95% CI** | **p-value** |
| --- | --- | --- | --- | --- |
| Precision | 0.9464 (0.8852, 0.9728) | 0.9194 (0.8243, 0.9562) | (0.0065, 0.0439) | 0.0071 |
| Recall | 0.5847 (0.2393, 0.7718) | 0.6902 (0.4046, 0.8157) | (-0.1626, 0.0088) | 0.1009 |
| F1 Score | 0.6987 (0.3820, 0.8419) | 0.7675 (0.5646, 0.8548) | (-0.1044, 0.0192) | 0.2132 |
| IoU | 0.5370 (0.2363, 0.7269) | 0.6227 (0.3934, 0.7464) | (-0.1375, 0.0251) | 0.2132 |
| Dice-Coefficient | 0.6987 (0.3820, 0.8419) | 0.7675 (0.5646, 0.8548) | (-0.1044, 0.0192) | 0.2132 |
| Specificity | 1.0000 (1.0000, 1.0000) | 1.0000 (1.0000, 1.0000) | (0, 0) | 0.1999 |
| MCC | 0.7157 (0.4748, 0.8444) | 0.7815 (0.6145, 0.8591) | (-0.0919, 0.0208) | 0.2446 |

Summary descriptives table for lung cancer, and other cancer in validation set

| Metrics | **Lung cancer (n=93)** | **Other cancer(n=79)** | **95% CI** | **p-value** |
| --- | --- | --- | --- | --- |
| Precision | 0.9164 (0.8222, 0.9507) | 0.9528 (0.8946, 0.9775) | (-0.0493, -0.013) | 0.0006 |
| Recall | 0.6304 (0.3390, 0.8026) | 0.6862 (0.3430, 0.8078) | (-0.1024, 0.0509) | 0.5655 |
| F1 Score | 0.7428 (0.4865, 0.8439) | 0.7571 (0.5015, 0.8730) | (-0.0798, 0.0281) | 0.3486 |
| IoU | 0.5908 (0.3214, 0.7300) | 0.6091 (0.3346, 0.7746) | (-0.1076, 0.0345) | 0.3486 |
| Dice-Coefficient | 0.7428 (0.4865, 0.8439) | 0.7571 (0.5015, 0.8730) | (-0.0798, 0.0281) | 0.3486 |
| Specificity | 1.0000 (1.0000, 1.0000) | 1.0000 (1.0000, 1.0000) | (-1e-04, 0) | 0.26 |
| MCC | 0.7662 (0.5251, 0.8478) | 0.7732 (0.5703, 0.8751) | (-0.0775, 0.022) | 0.2686 |
